# Supplementary material for: Structures of channelrhodopsin paralogs in peptidiscs explain their contrasting K+ and Na+ selectivities
Source: Nat Commun. 2023 Jul 20;14:4365. doi: 10.1038/s41467-023-40041-2 (PMC10359266; doi:10.1038/s41467-023-40041-2)
Supplement: Supplementary file 1 — Supplementary Information [file 41467_2023_40041_MOESM1_ESM.pdf]

Supplementary Information

Structures of channelrhodopsin paralogs in peptidiscs explain their contrasting K^+ and Na^+ selectivities

Morizumi *et al.*

This PDF file includes:

Supplementary Figures

Supplementary Discussion

Supplementary References

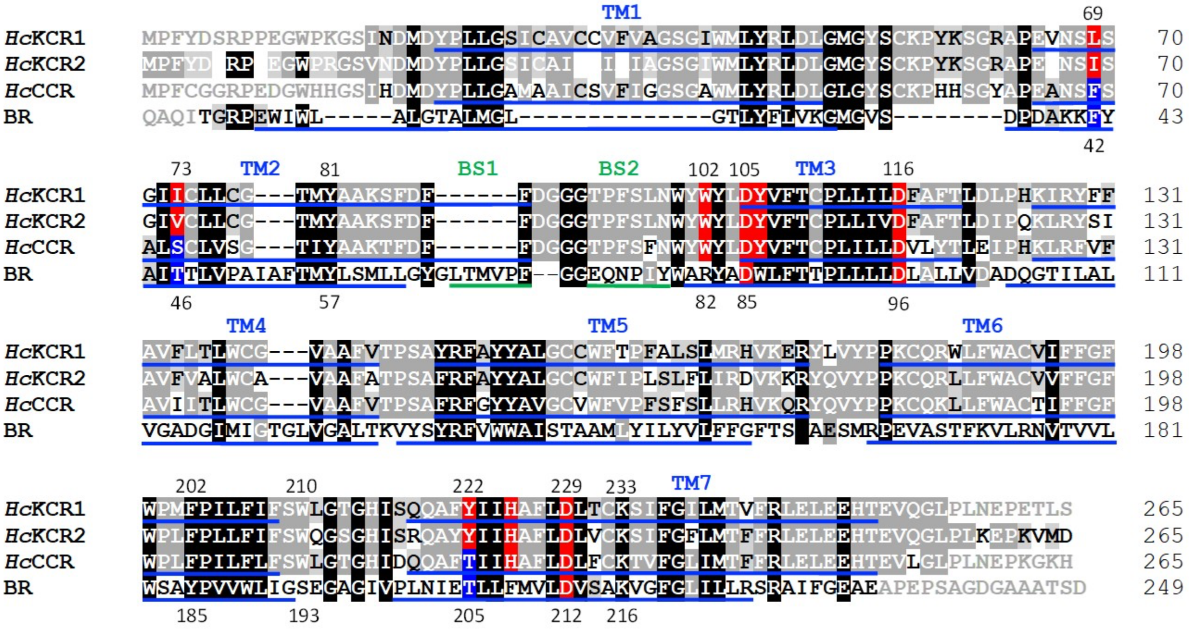

Supplementary Fig. 1 | The protein alignment of the 7TM domains of *Hyphochytrium catenoides* channelrhodopsins and *Halobacterium salinarum* bacteriorhodopsin. Transmembrane helices (TM1-TM7) are underlined in blue as defined in the PDB IDs: 8GI8 for *Hc*KCR1, 8GI9 for *Hc*CCR, and 7Z09 for BR, respectively. The two beta strands (BS) found in BR are underlined in green. Residues not resolved in the structures are shown in grey. The sequence of *Hc*KCR2 is shown for comparison. Functionally important residues discussed in the text are highlighted in red (conserved in *Hc*KCR1) and blue (conserved in *Hc*CCR). The residue numbers on the top correspond to *Hc*KCR1 and *Hc*CCR, on the bottom, to BR. The numbers on the right show the last residue number in each line. The gaps in TM1 and TM2 alignment are due to a low degree of residue conservation between *Hc*ChRs and BR.

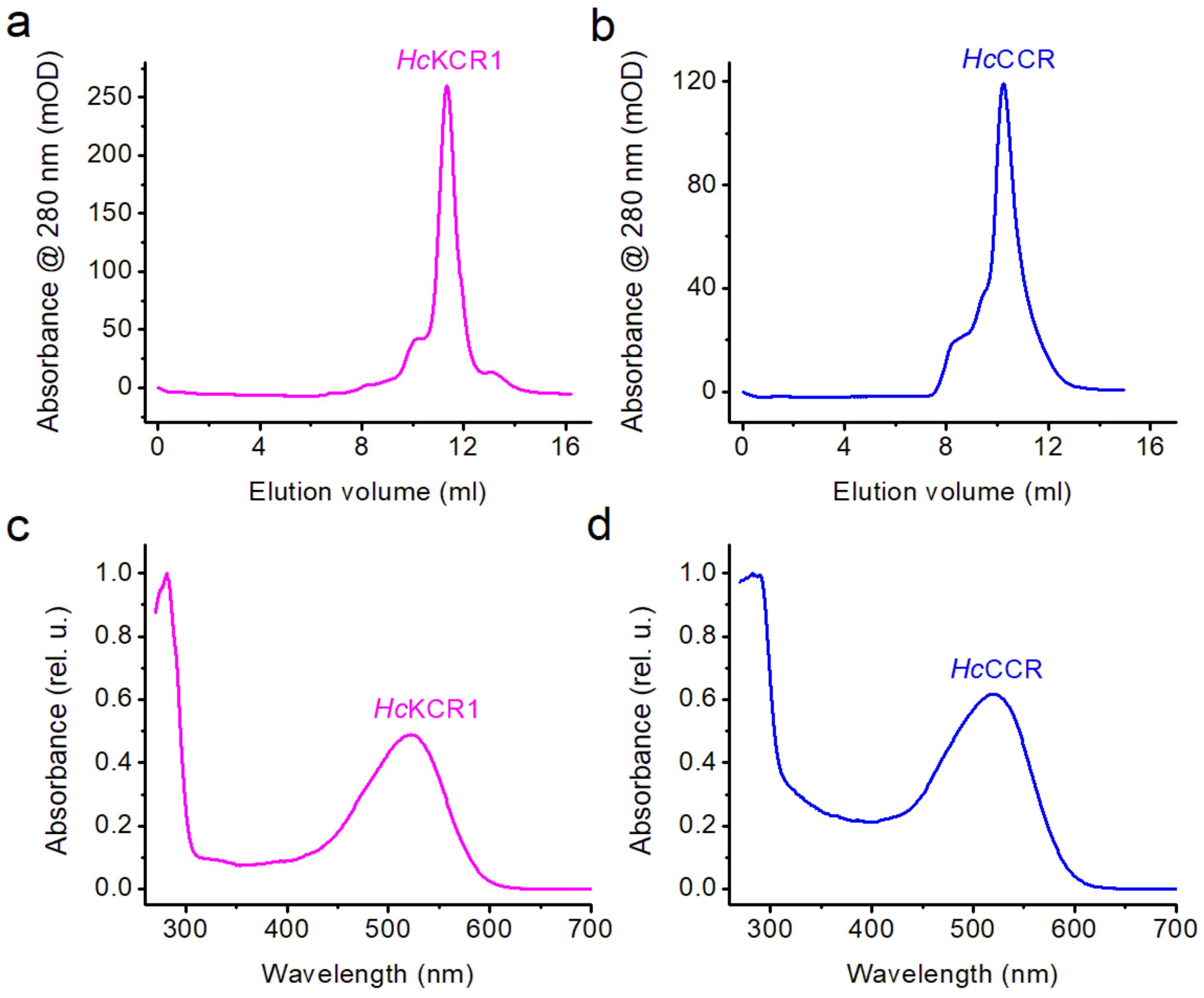

Supplementary Fig. 2 | Size exclusion chromatography (SEC) and UV/vis spectra of purified *Hc*KCR1 and *Hc*CCR. **a, b** Representative SEC profiles at 280 nm of *Hc*KCR1 and *Hc*CCR, respectively. **c, d** UV/vis spectra of purified proteins.

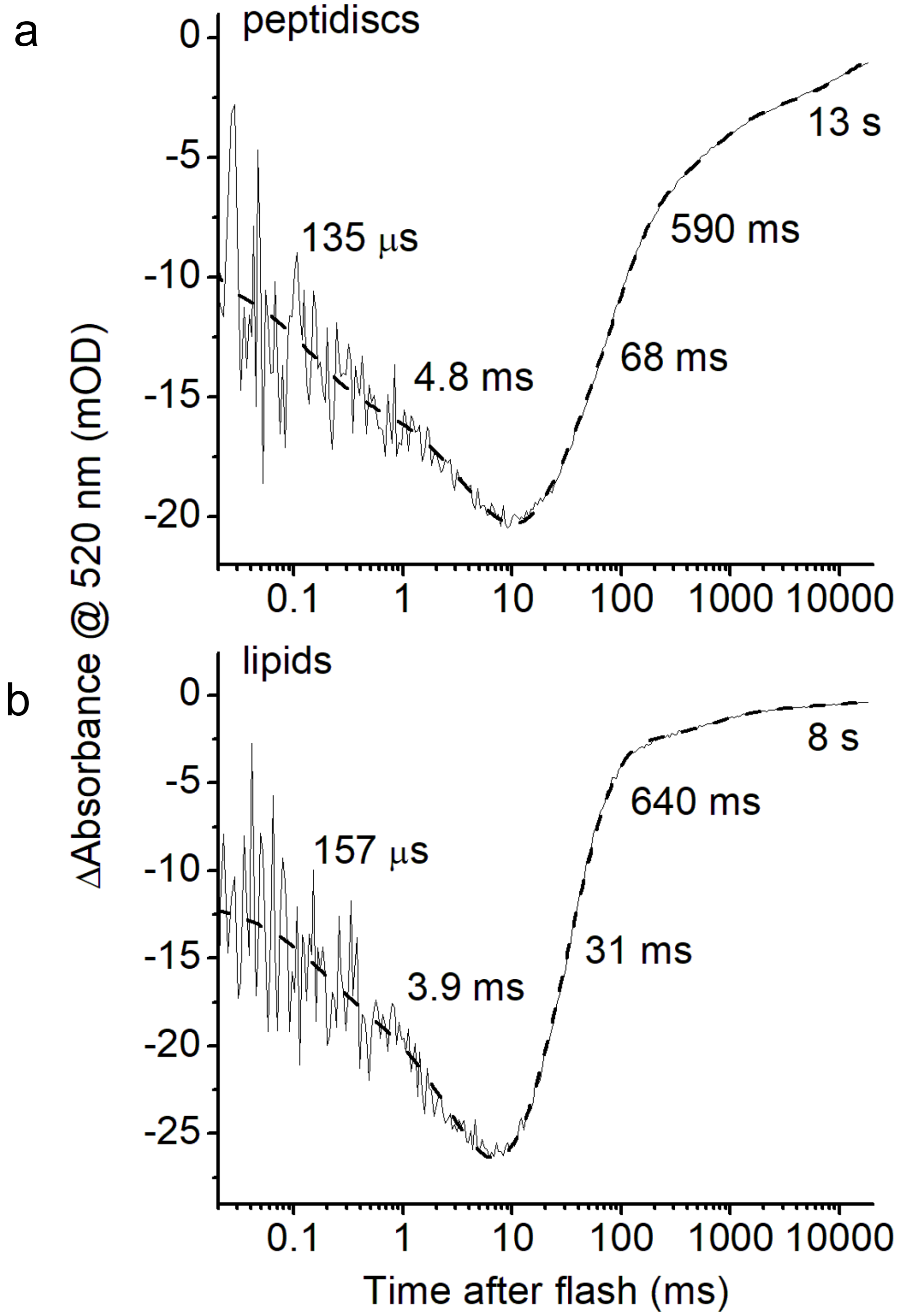

Supplementary Fig. 3 | Absorption changes of *Hc*KCR1 in response to 532-nm laser flashes. **a** *Hc*KCR1 reconstituted into peptidiscs. **b** *Hc*KCR1 reconstituted into liposomes. The thin solid lines are experimental data, the thick dashed lines, multiexponential computer approximations. The numbers are the time constants of individual kinetic components. For details see Methods.

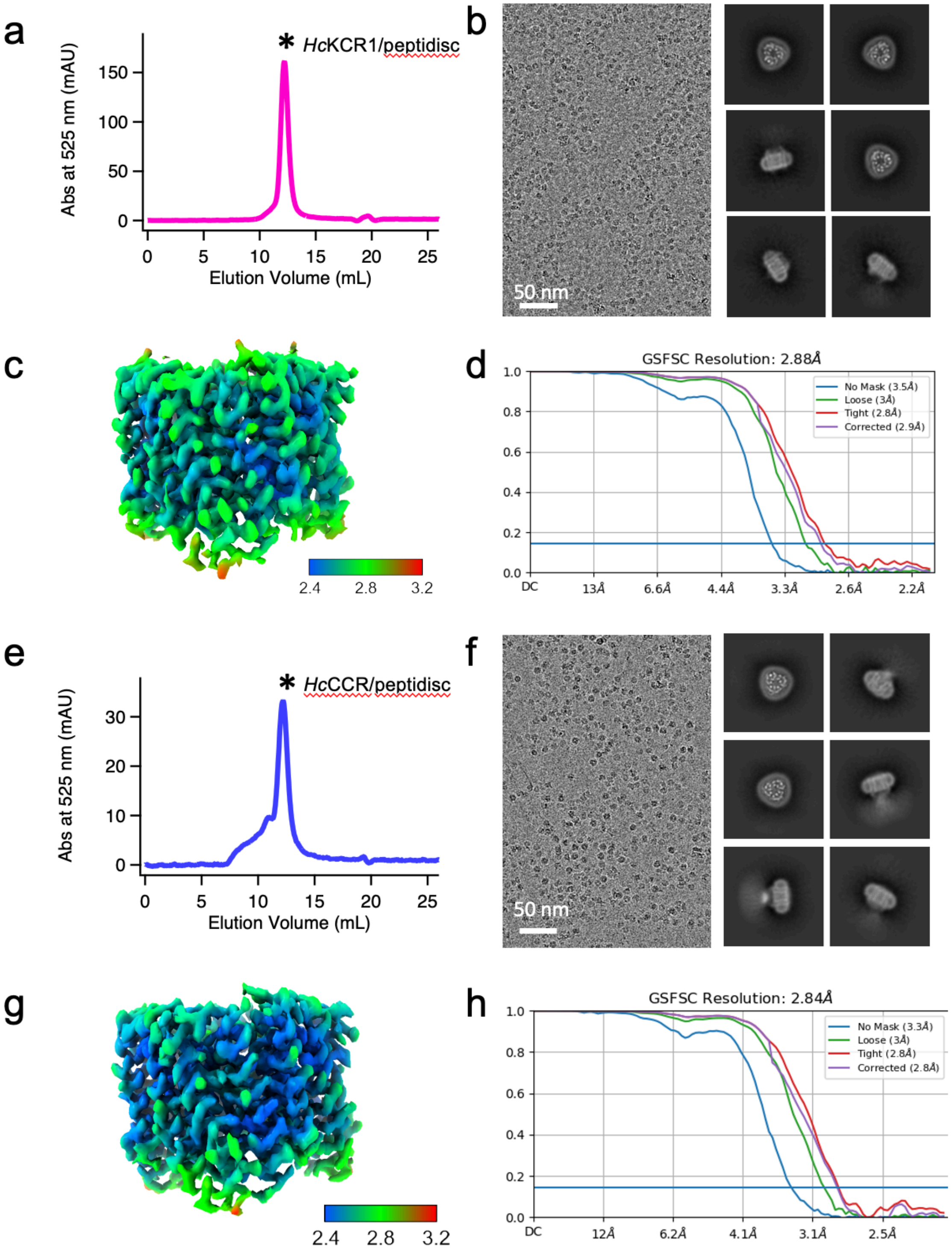

Supplementary Fig. 4 | Cryo-EM analysis of *Hc*KCR1 and *Hc*CCR. a Size exclusion chromatography profile for *Hc*KCR1 after reconstitution into peptidiscs. **b** Representative cryo-EM micrograph and 2D classes (see also Methods and Supplementary Fig. 5). **c** Local resolution map. **d** Gold standard Fourier shell correlation (FSC) curve and global resolution estimation. Local resolution maps and FSC curves were generated with cryoSPARC v4.1. **e-h** same as **a-d** for *Hc*CCR (see also Methods and Supplementary Fig. 6).

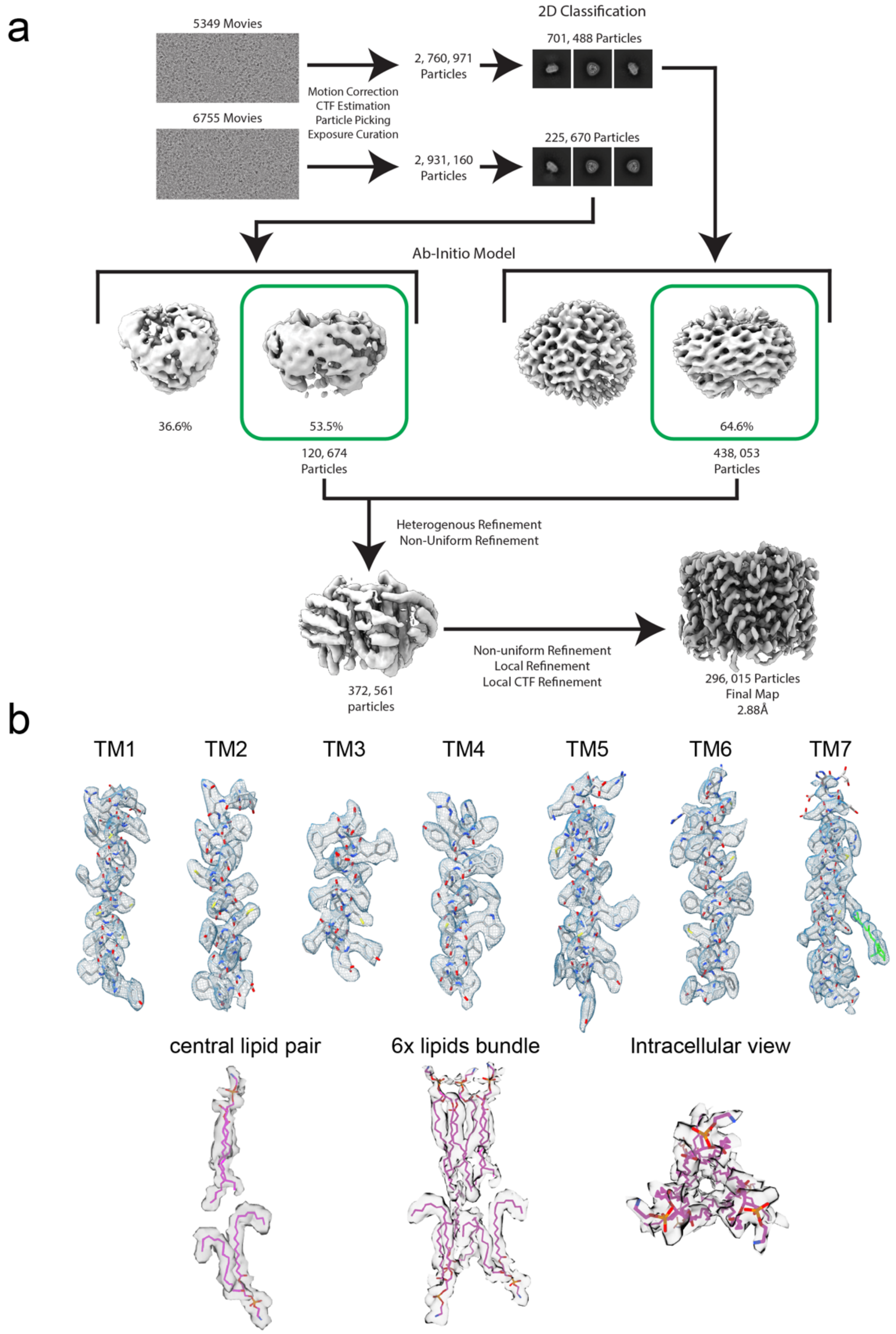

Supplementary Fig. 5 | Cryo-EM data processing for *Hc*KCR1. a Workflow. Initial data processing and refinement of electron density maps were performed in cryoSPARC v4.1. Two separate datasets were merged after initial 3D reconstruction followed by 3D refinement to generate the final map. **b** Representative density maps with fitted models for transmembrane regions and internal lipids within the structure.

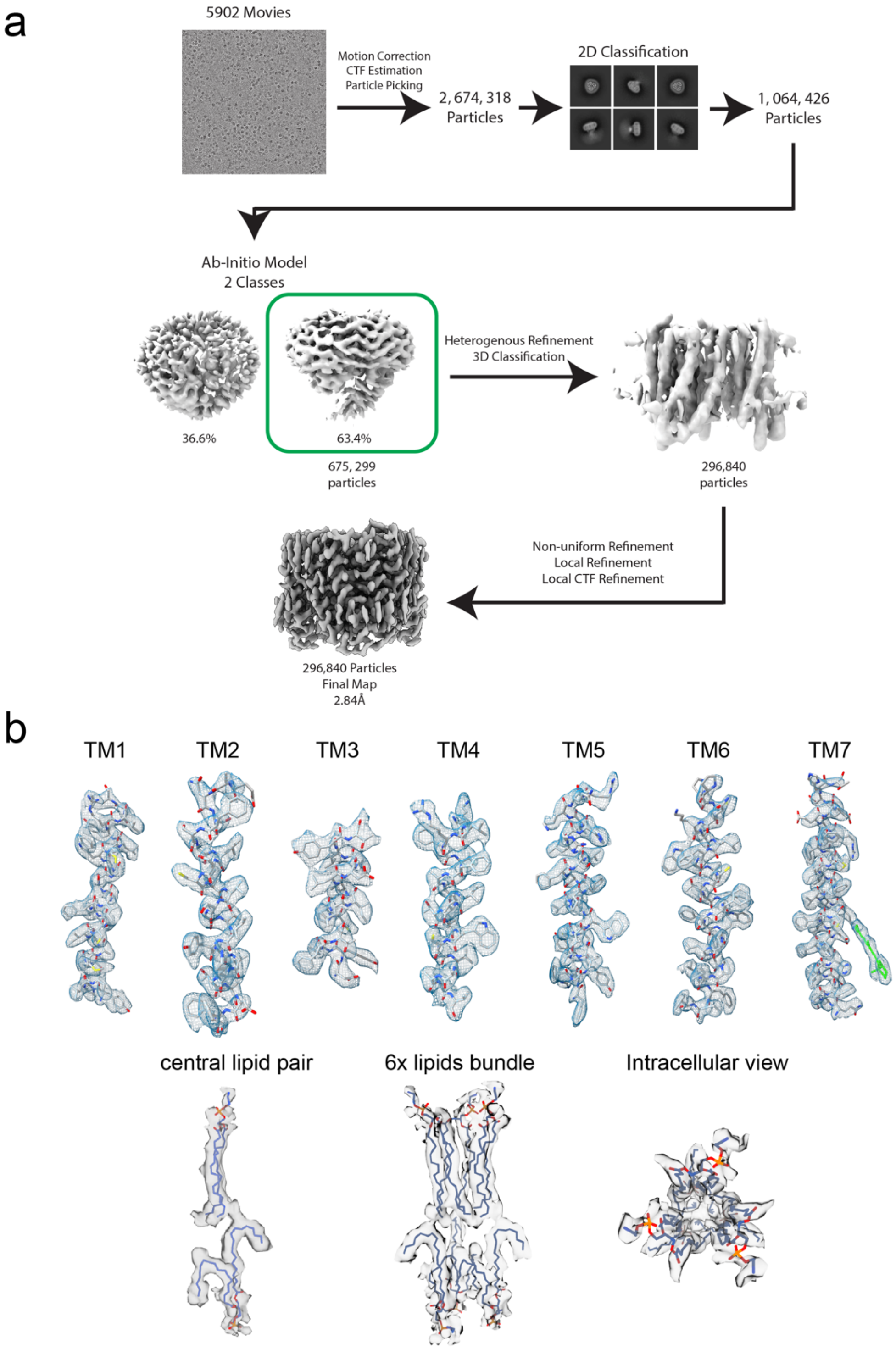

Supplementary Fig. 6 | Cryo-EM data processing for *Hc*CCR. a Workflow. Initial data processing and refinement of electron density maps were performed in cryoSPARC v4.1. **b** Representative density maps and fitted models for transmembrane regions and internal lipids within the structure.

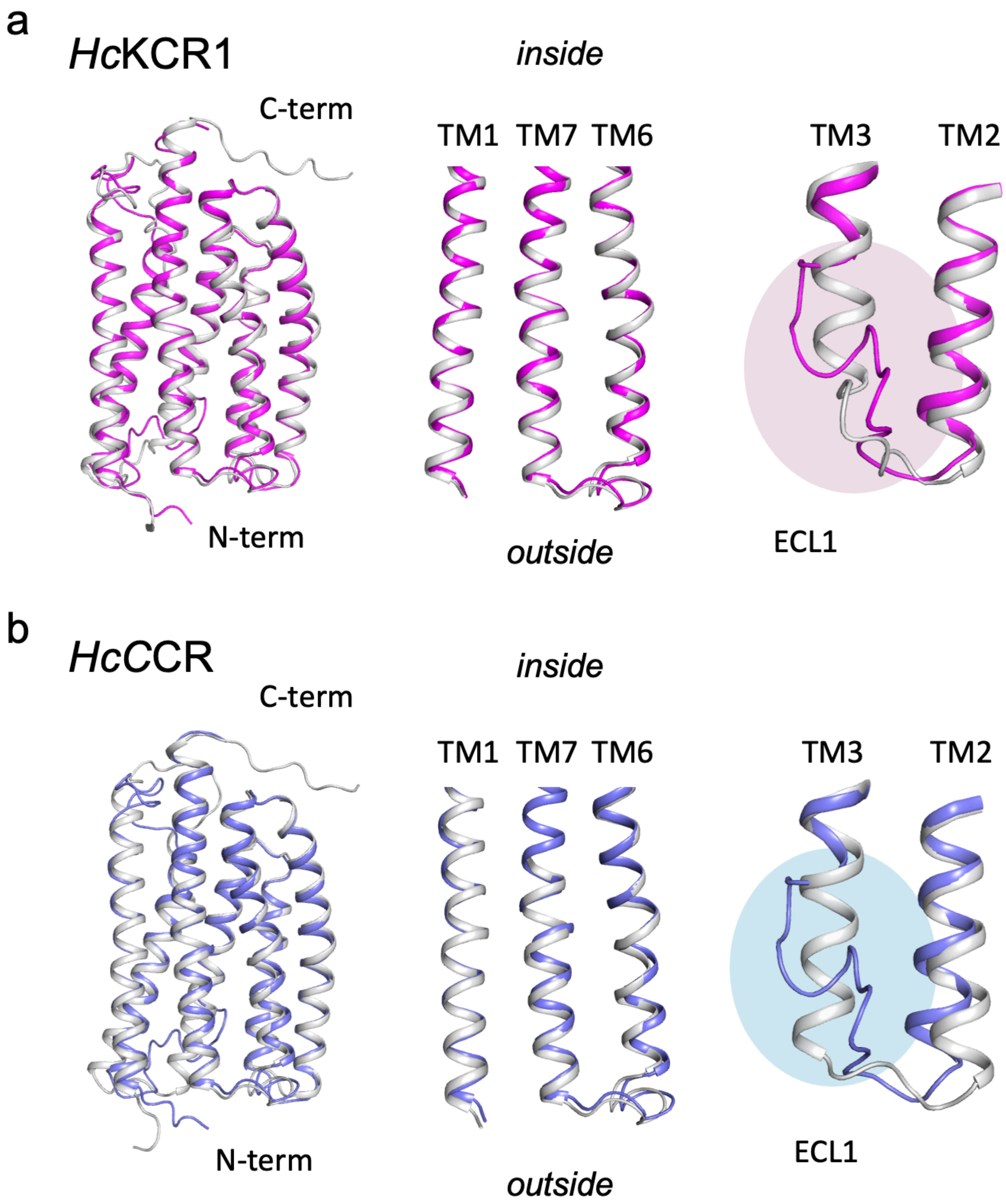

Supplementary Fig. 7 | Comparison of cryo-EM structures with previously published homology models[1]. a *Hc*KCR1 (pink cartoon). **b** *Hc*CCR (blue cartoon). Overlay of structures (pink or blue) with homology model (white cartoon). Good agreement is seen for TM1, TM7 and TM6. For the extracellular TM2-TM3 loop (ECL1) the cryo-EM structures reveal partial unwinding of the extracellular end of TM3.

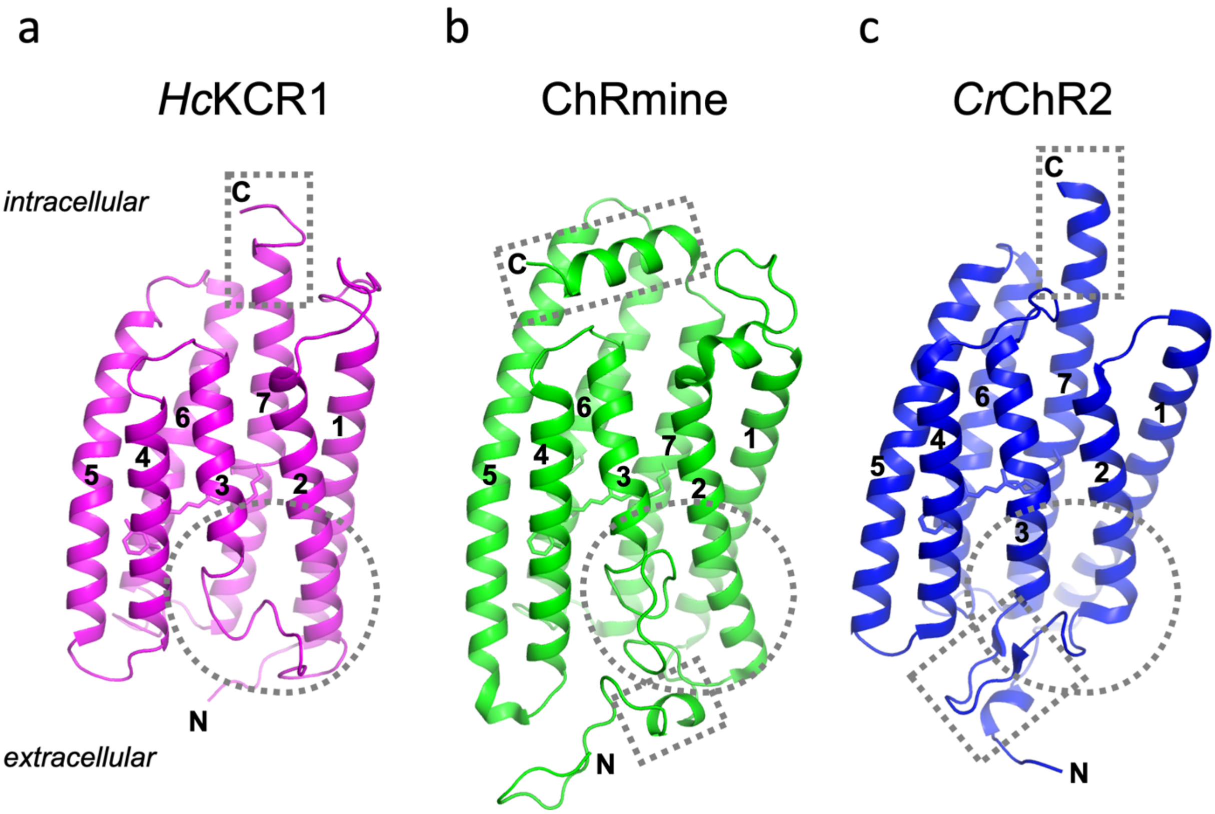

Supplementary Fig. 8 | Comparison of overall protomer structures of *Hc*KCR1, ChRmine and *Cr*ChR2. a *Hc*KCR1 (magenta, PDB ID: 8GI8), **b** ChRmine (green, PDB ID: 7SFK), and **c** *Cr*ChR2 (blue, PDB ID: 6EID). TM helices are numbered 1-7. Regions with main differences are highlighted by broken lines. For details see Supplementary Discussion.

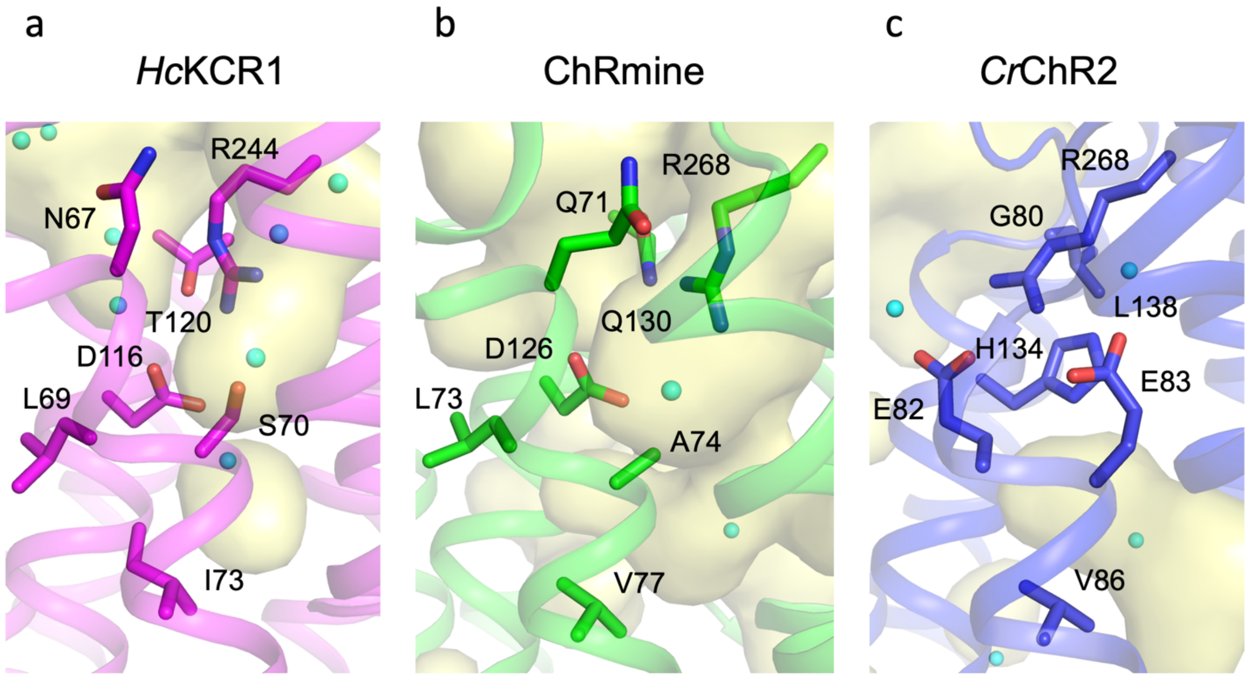

Supplementary Fig. 9 | Comparison of intracellular segments of *Hc*KCR1, ChRmine and *Cr*ChR2. a *Hc*KCR1 (magenta, PDB ID: 8GI8), **b** ChRmine (green, PDB ID: 7SFK), and **c** *Cr*ChR2 (blue, PDB ID: 6EID). The internal cavities modelled with the program HOLLOW[2] are shown. Water molecules are shown as spheres. For details see Supplementary Discussion.

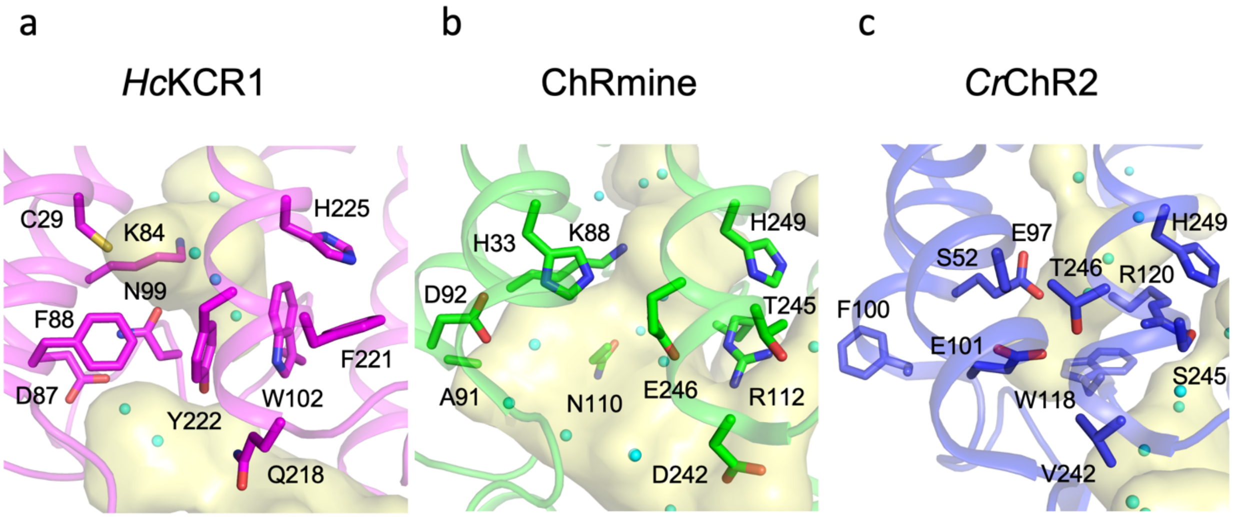

Supplementary Fig. 10 | Comparison of extracellular segments of *Hc*KCR1, ChRmine and *Cr*ChR2. a *Hc*KCR1 (magenta, PDB ID: 8GI8), **b** ChRmine (green, PDB ID: 7SFK), and **c** *Cr*ChR2 (blue, PDB ID: 6EID). The internal cavities modelled with the program HOLLOW[2] are shown. Water molecules are shown as spheres. For details see Supplementary Discussion.

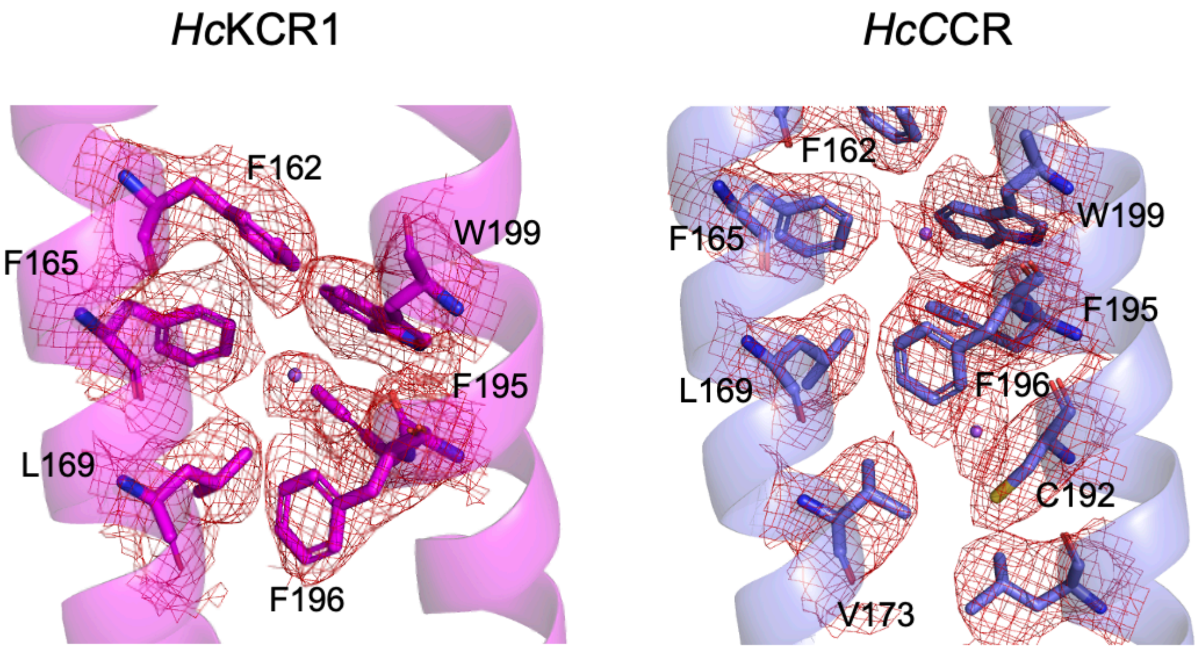

Supplementary Fig. 11 | Sodium ions and surrounding residues in *Hc*KCR1 and *Hc*CCR. Shown are Na^+ ions as spheres and residues as stick models, as well as densities.

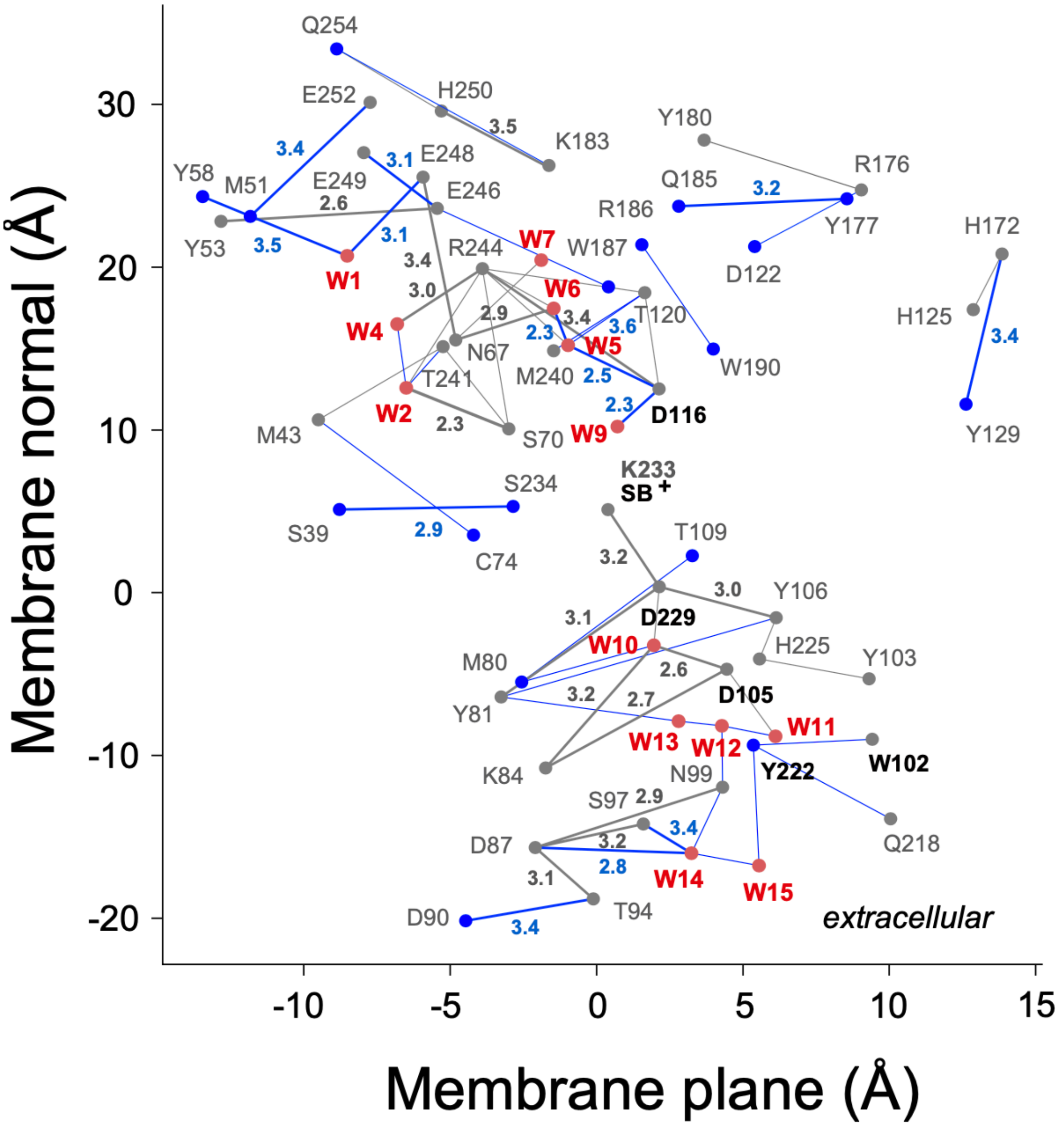

Supplementary Fig. 12 | H-bond graph of *Hc*KCR1 as in Fig. 3c. Distances of H-bonds are given in Å and are derived from PDB ID: 8GI8. For clarity, only selected distances between amino acid residue side chains are indicated in the graph.

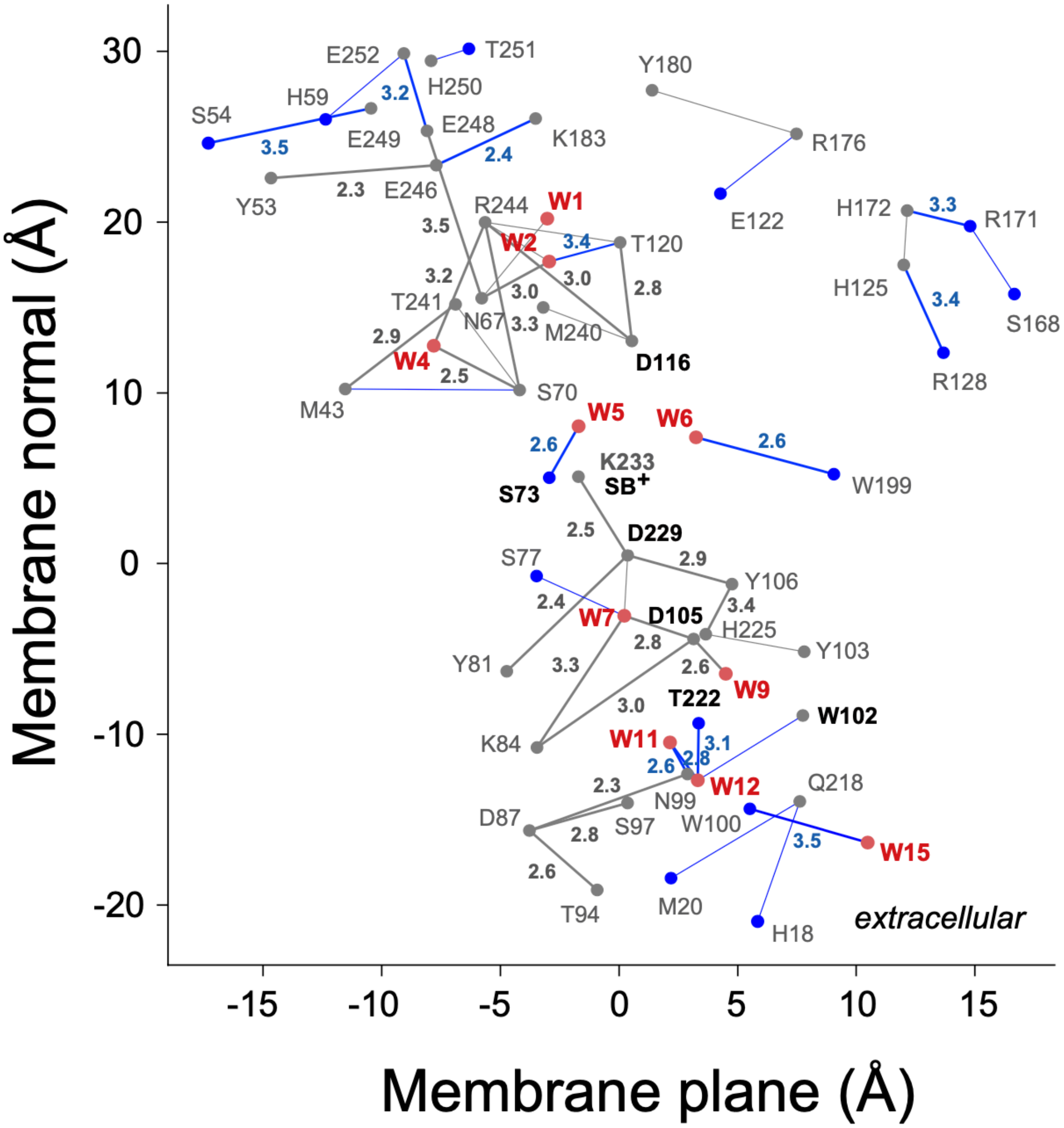

Supplementary Fig. 13 | H-bond graph of *Hc*CCR as in Fig. 3d. Distances of H-bonds are given in Å and are derived from PDB ID: 8GI9. For clarity, only selected distances between amino acid residue side chains are indicated in the graph.

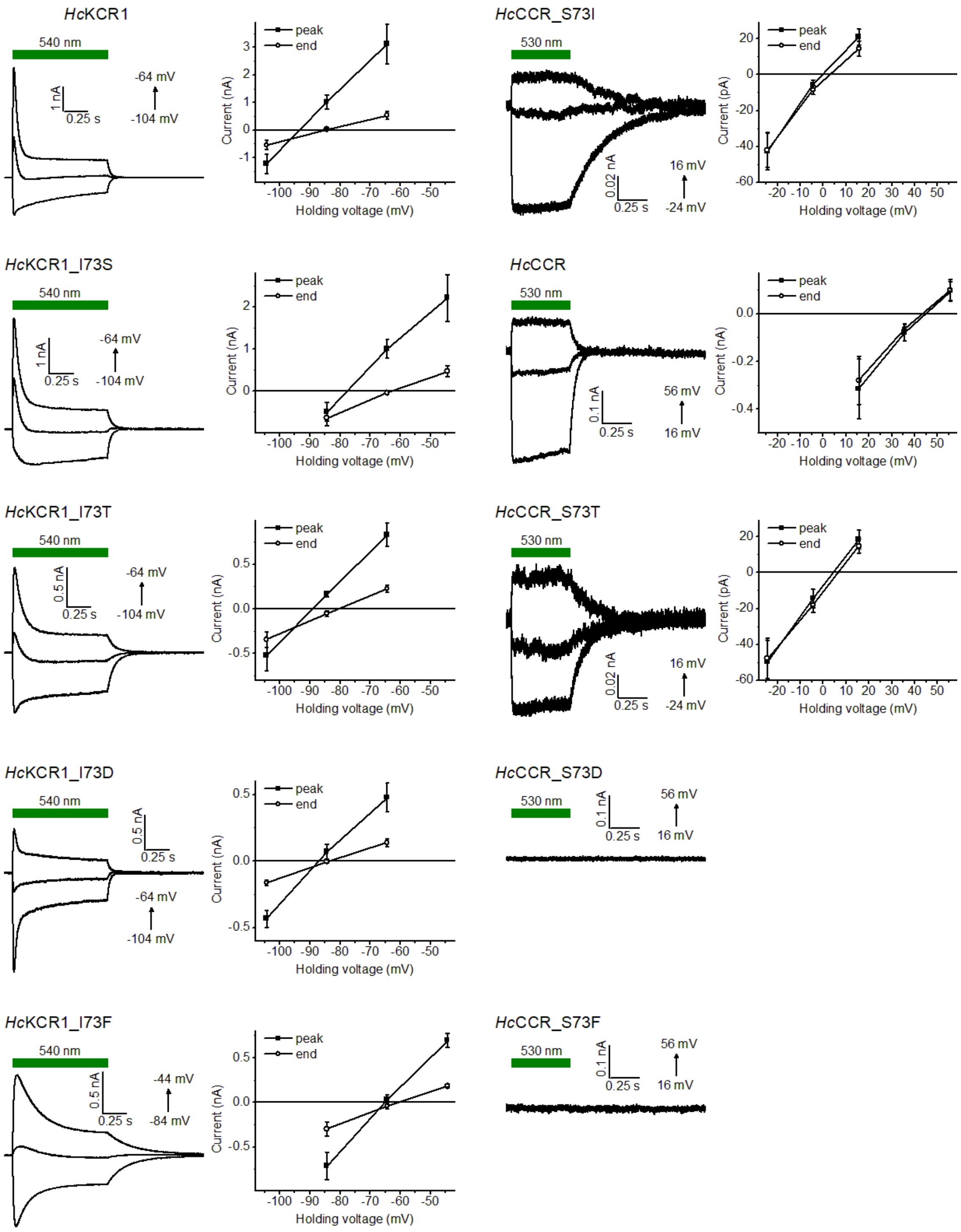

Supplementary Fig. 14 | Photocurrent traces and current-voltage relationships (IV curves) of the mutants at the residue 73 position. The green bars represent the duration of illumination. The holding voltage (V_h) was changed in 20-mV increments. The V_h values were corrected for the liquid junction potentials (LJPs). The symbols show the mean values, the error bars, s.e.m. (n = 8 cells for *Hc*KCR1_I73S and WT *Hc*CCR, and 7 cells for all other variants). Source data are provided as a Source Data file.

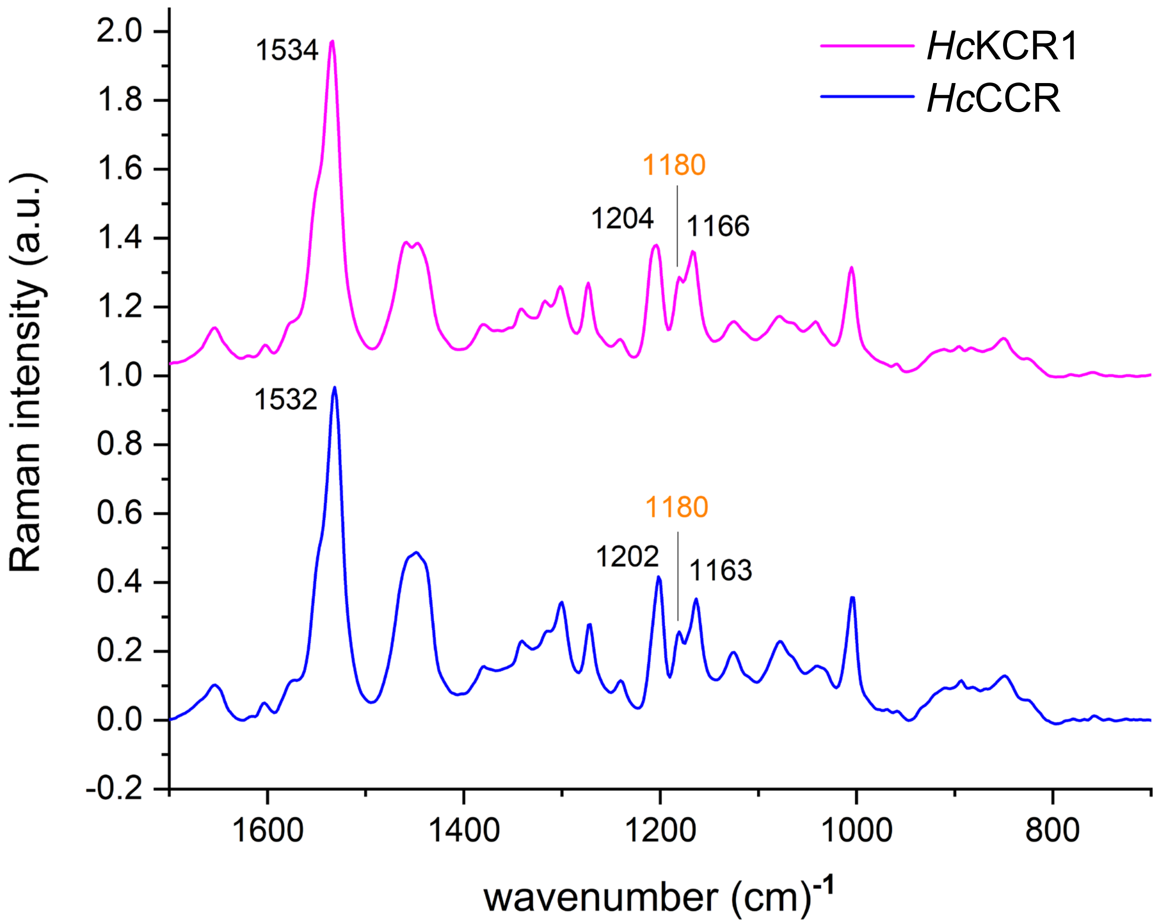

Supplementary Fig. 15 | FT-Raman spectra of solubilized *Hc*KCR1 (magenta) and *Hc*CCR (blue), vertically offset for clarity. The ethylenic C=C stretches and fingerprint C-C stretches are labeled with the corresponding wavenumbers. The 1180 cm^{-1} band characteristic of 13-*cis*-retinal is highlighted with the orange label.

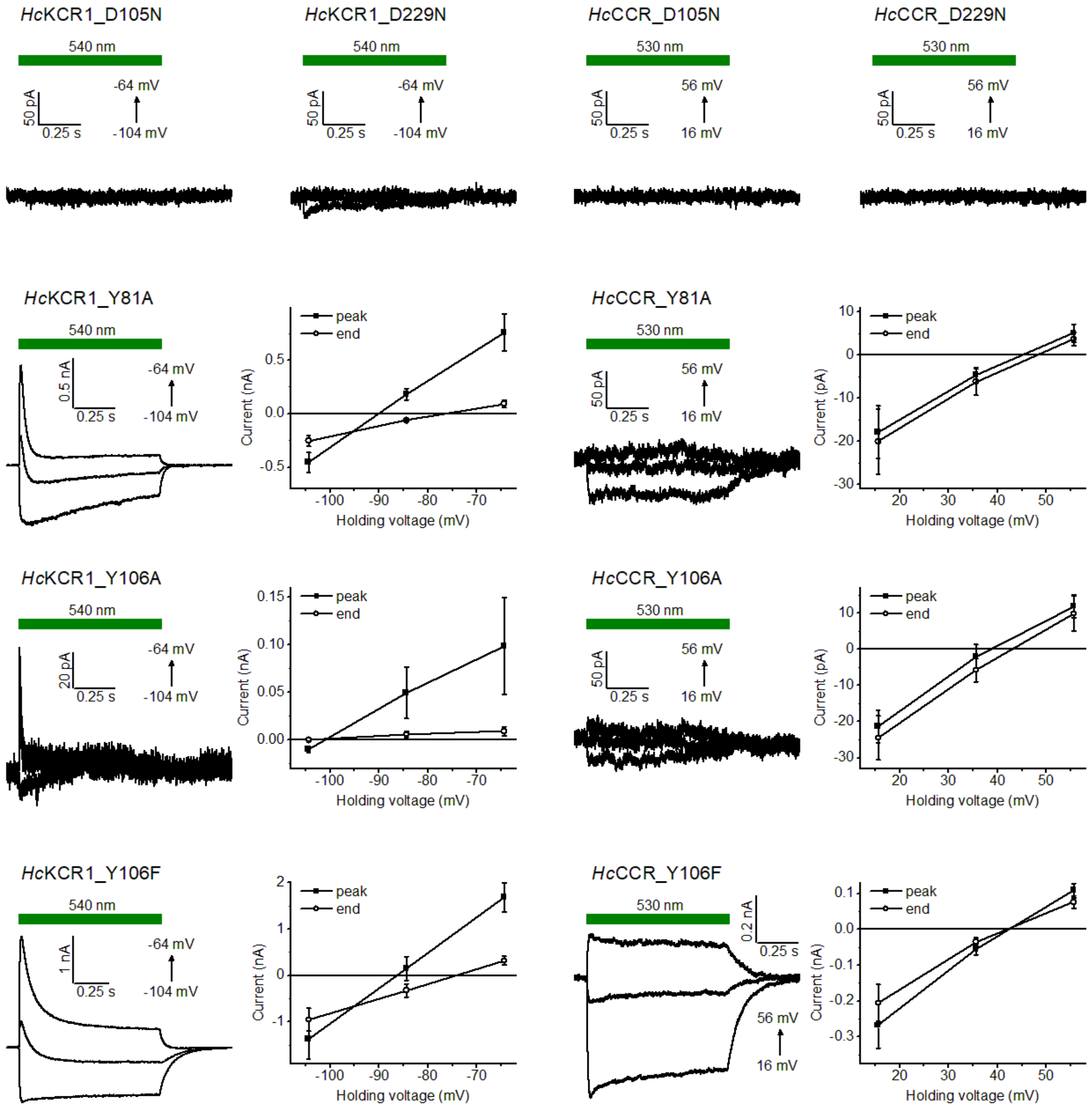

Supplementary Fig. 16 | Photocurrent traces and current-voltage relationships (IV curves) of the photoactive site mutants. The green bars represent the duration of illumination. The holding voltage (V_h) was changed in 20-mV increments. The V_h values were corrected for the liquid junction potentials (LJPs). The symbols show the mean values, the error bars, s.e.m. (n = 8 cells for *Hc*KCR1_Y106F and 7 cells for all other variants). Source data are provided as a Source Data file.

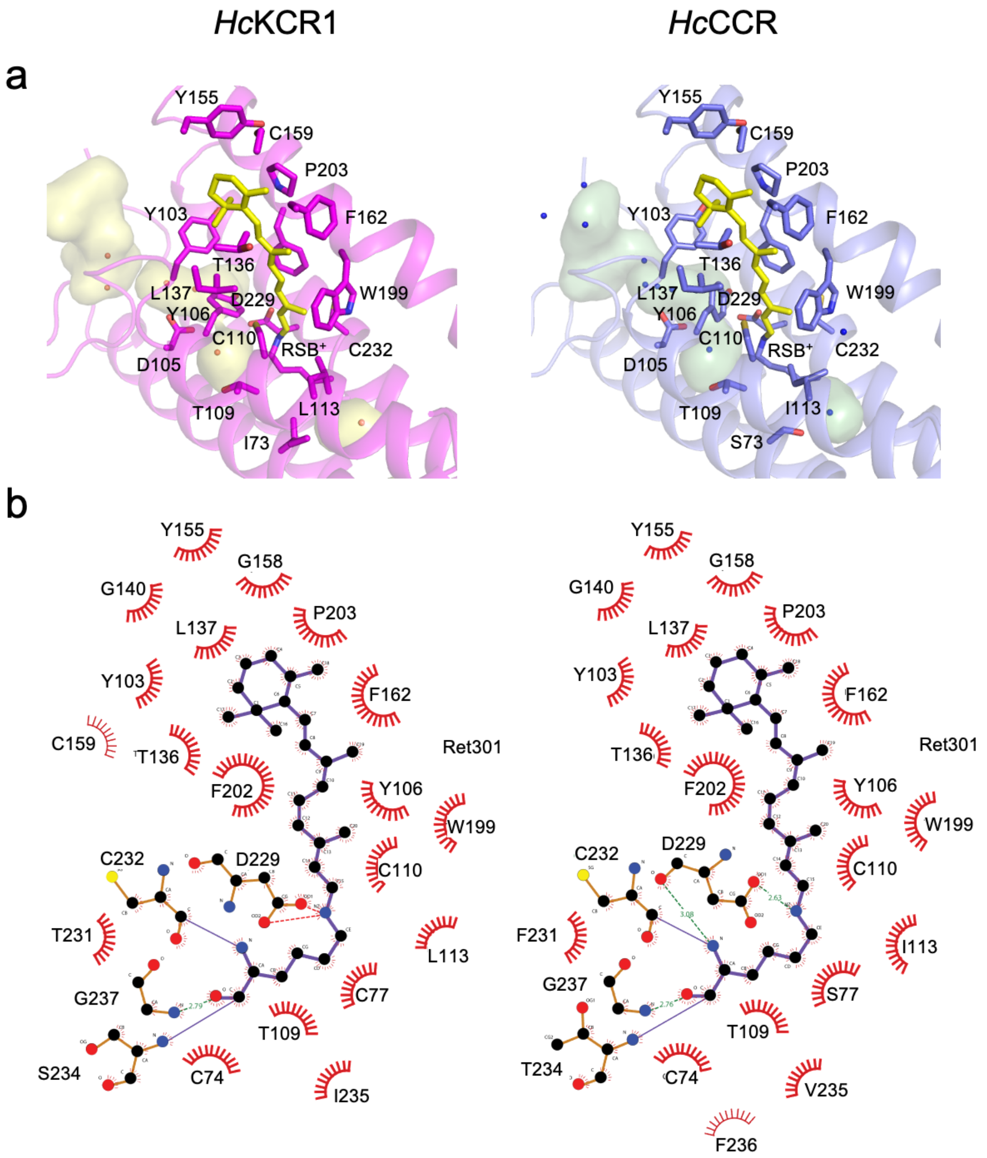

Supplementary Fig. 17 | Retinal binding pocket. a Comparison of the local environment of the retinal binding pocket. Retinal is displayed in yellow, with key residues labelled. Nearby cavities are displayed with a surface representation. **b** 2D schematic of binding pocket. Retinal and the Schiff base lysine are colored in black, with nearby residues represented as red circles. Schematics were generated with LigPlot^{+} [3].

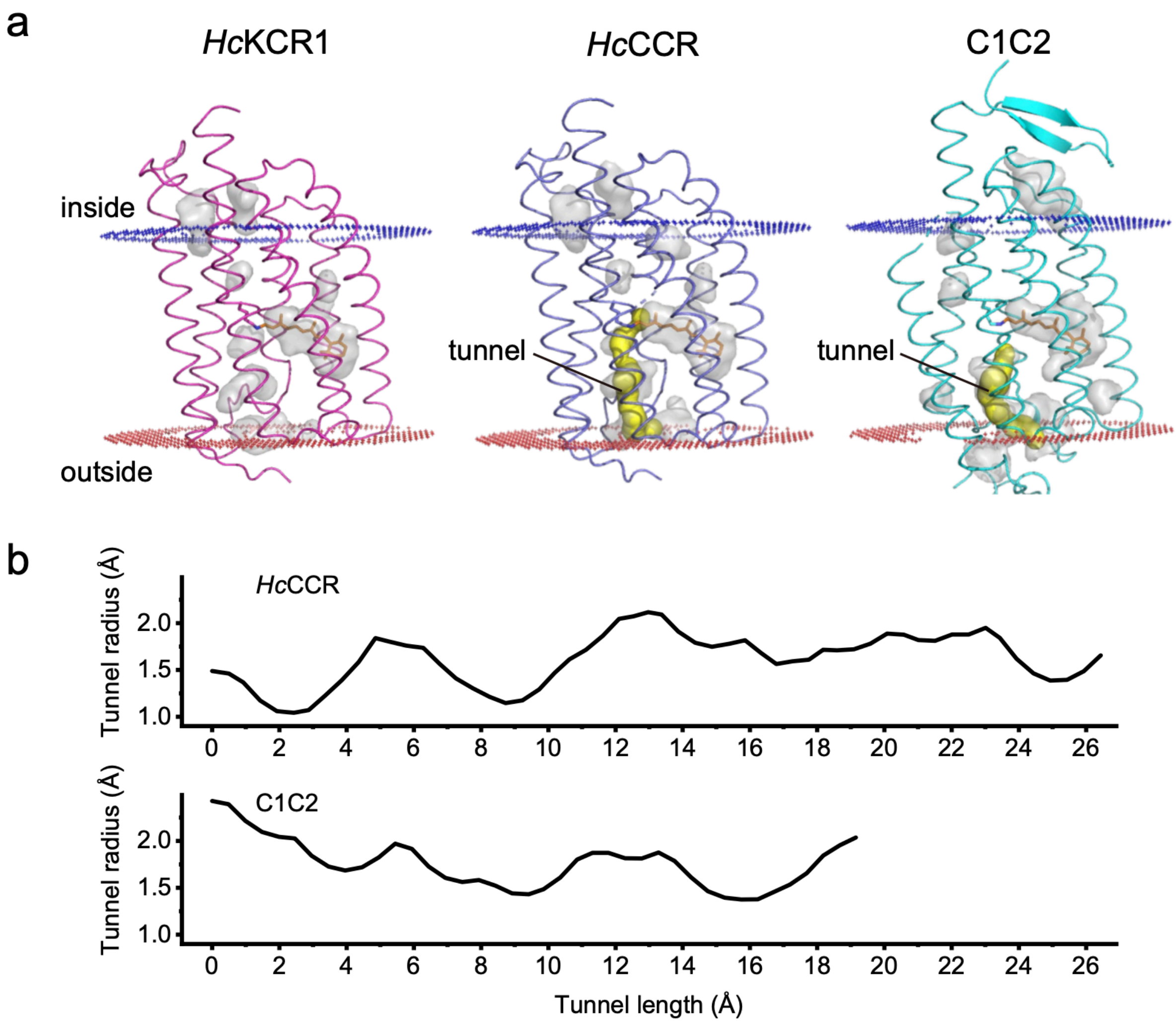

Supplementary Fig. 18 | Detection of intramolecular tunnels by CAVER[4]. a ChR structures showing the helices (magenta, purple and cyan for *Hc*KCR1, *Hc*CCR and C1C2 (PDB ID: 3UG9), respectively); the internal cavities (gray); the tunnel detected with the probe radius 0.9 Å (yellow); the retinal chromophore (orange); the cytoplasmic and extracelullular surfaces of the membrane (blue and red, respectively). **b** The tunnel profiles in *Hc*CCR and C1C2. No tunnel could be detected in *Hc*KCR1.

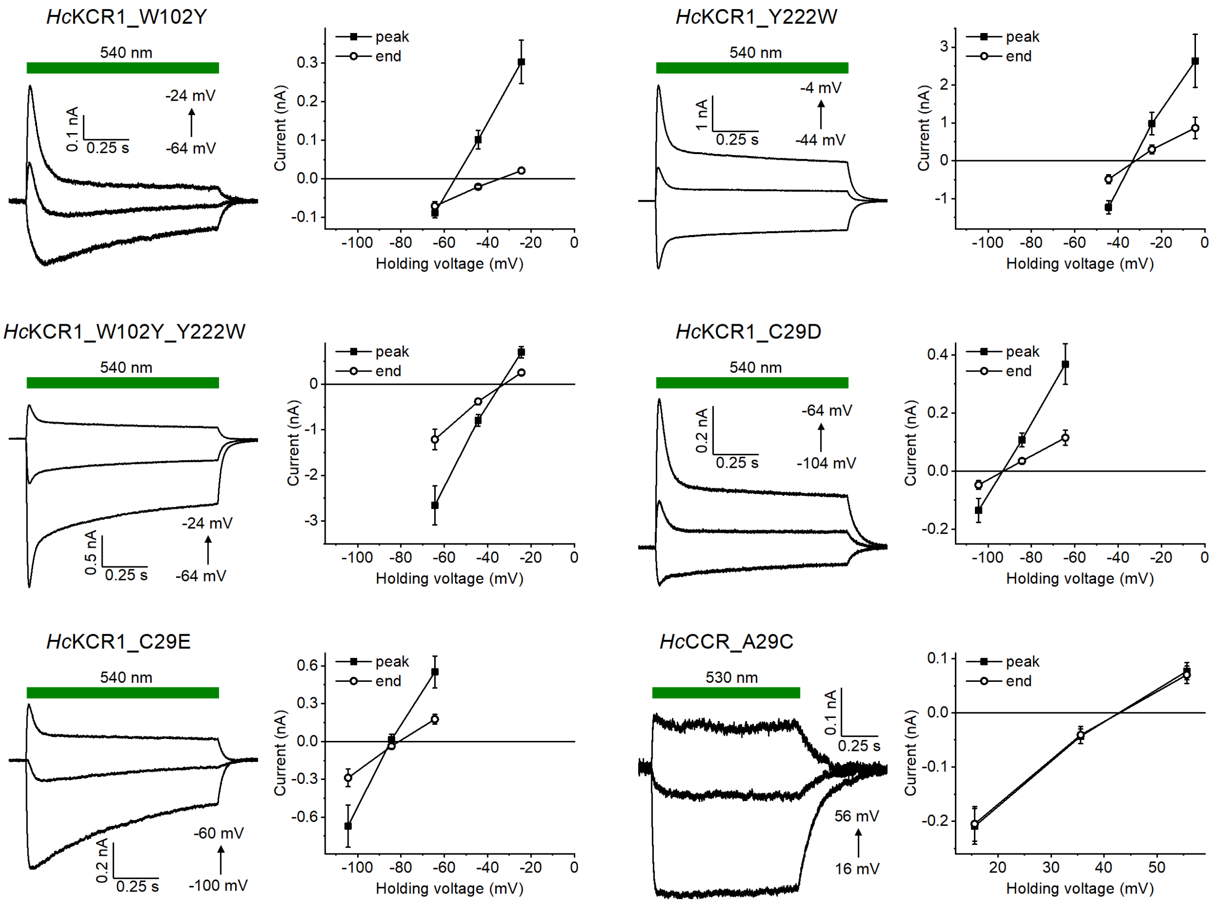

Supplementary Fig. 19 | Photocurrent traces and current-voltage relationships (IV curves) of the mutants in the extracellular segment of the cation conduction pathway. The green bars represent the duration of illumination. The holding voltage (V_h) was changed in 20-mV increments. The V_h values were corrected for the liquid junction potentials (LJPs). The symbols show the mean values, the error bars, s.e.m. (n = 7 cells). Source data are provided as a Source Data file.

Supplementary Discussion

Comparison of *Hc*KCR1 and *Hc*CCR with other CCR structures

Consistent with their sequence homology (Supplementary Fig. 1), *Hc*ChRs share their trimeric assembly with ChRmine (*Rhodomonas lens* CCR1), the only representative of cryptophyte BCCRs with known high resolution cryo-EM structure[5, 6]. As mentioned in the main text, the residues that form interprotomer contacts in ChRmine are not conserved in *Hc*ChRs. Within an individual protomer, a common feature between *Hc*ChRs and ChRmine is partial unwinding of TM3 at the extracellular side, not found in other microbial rhodopsins, although in *Hc*ChRs this unwound region and the TM2-TM3 loop adopt different conformations compared to ChRmine (a comparison of *Hc*KCR1, ChRmine, and *Cr*ChR2 structures is shown in Supplementary Fig. 8; the differences between *Hc*KCR1 and *Hc*CCR are discussed in the main text). Also, *Hc*ChRs do not contain α-helical regions in either the N- or C-terminal segments as does ChRmine.

In contrast to *Hc*ChRs and ChRmine, all chlorophyte CCRs with available structures are dimers[7, 8, 9]. Supplementary Figs. 8a, c show protomer comparison of *Hc*KCR1 with channelrhodopsin 2 from *Chlamydomonas reinhardtii* (*Cr*ChR2)[10], the best studied chlorophyte CCR. The N-terminus of *Hc*KCR1 is shorter and lacks the additional α-helical region found in *Cr*ChR2. TM1 of *Hc*KCR1 does not protrude as much into the intracellular space, and is less tilted towards the membrane plane than that of *Cr*ChR2. The TM1-TM2 loop and TM2 are longer in *Hc*KCR1 than in *Cr*ChR2, but the largest difference is observed in the TM2-TM3 loop that in *Hc*KCR1 lacks a β-sheet, and in TM3 that in *Hc*KCR1 is partially unwound. The TM3-TM4 loop, TM4, TM5 and TM7 are all shorter in *Hc*ChRs than in *Cr*ChR2.

In the intracellular segment of the putative cation conduction pathway (Supplementary Fig. 9), the residues contributing to K^+ selectivity in *Hc*KCR1 (Leu69, Ile73 and Asp116) are conserved or replaced with structurally similar residues in ChRmine (Leu73, Val77 and Asp126, respectively). As shows our analysis of KCR homologs, this residue combination is not sufficient for K^+ selectivity[1]. In *Cr*ChR2, Leu69 is replaced with Glu82, which, together with Glu83, His134 and Arg268 (in *Hc*KCR1 corresponding to Ser70, Asp116 and Arg244, respectively) form the so-called "inner gate"[8]. Glu83 is the key component of this gate and is required for cation selectivity of *Cr*ChR2[11]. Glu83 is H-bonded to H134, and the presence of a non-carboxylate residue in the position of Asp116/Asp126 (*Hc*KCR1/ChRmine) is characteristic of chlorophyte CCRs in contrast to cryptophyte BCCRs and KCR homologs. Replacement of Glu83 with smaller non-carboxylate residues in *Hc*KCR1 and ChRmine (Ser70 and Ala74, respectively) helps to avoid electrostatic repulsion by Asp116/Asp126. Arg244 that forms a salt bridge with Asp116 in *Hc*KCR1 and is predicted to flip upon opening of the channel[12], is conserved in both ChRmine and *Cr*ChR2, despite their relatively low overall sequence homology. Thus this Arg appears to be an important determinant of cation channel function, although it can be replaced with other residues (mostly Lys or Gln) in other CCRs and BCCRs.

In the extracellular segment (Supplementary Fig. 10), the main difference between *Hc*KCR1 and ChRmine is replacement of hydrophobic residues (Phe88, Trp102, Phe221, and Tyr222) with charged or polar residues (Asp92, Arg112, Thr245, and Glu246, respectively; for sequence

alignments see Refs. [12, 13]). As described in the main text, this hydrophobic cluster and especially Tyr222 are major determinants of K^+ selectivity in *Hc*KCR1. His225 of *Hc*KCR1 is conserved not only in ChRmine (His249), but also in *Cr*ChR2 (His249), in which it contributes to the "extracellular gate"[8], together with Arg120 and Ser245, corresponding to Trp102 and Phe221 in *Hc*KCR1 and Arg112 and Thr245 in ChRmine. Again, *Hc*ChR's His225, as Arg244, is highly conserved in other CCR and BCCRs and appears to be a common element of their cation conductance mechanism. Some residues that are conserved in *Hc*KCR1 and ChRmine (e.g., Lys84/Lys88), nevertheless, adopt different conformations of their side chains. In *Hc*KCR1, the ε-amino group of Lys84 faces and H-bonds with Asp105 (Fig. 3c), whereas in ChRmine, that of Lys88 faces away from the corresponding Asp115[5, 6].

Supplementary References

1. Govorunova E. G., Sineshchekov, O. A., Brown, L. S., Bondar, A. N. & Spudich, J. L. Structural Foundations of Potassium Selectivity in Channelrhodopsins. *mBio* **13**, e0303922 (2022).
2. Ho B. K. & Gruswitz, F. HOLLOW: generating accurate representations of channel and interior surfaces in molecular structures. *BMC Struct Biol* **8**, 49 (2008).
3. Laskowski R. A. & Swindells, M. B. LigPlot+: multiple ligand-protein interaction diagrams for drug discovery. *J Chem Inf Model* **51**, 2778-2786 (2011).
4. Brezovsky J., Kozlikova, B. & Damborsky, J. Computational Analysis of Protein Tunnels and Channels. *Methods Mol Biol* **1685**, 25-42 (2018).
5. Tucker K., Sridharan, S., Adesnik, H. & Brohawn, S. G. Cryo-EM structures of the channelrhodopsin ChRmine in lipid nanodiscs. *Nat Commun* **13**, 4842 (2022).
6. Kishi K. E.*, et al.* Structural basis for channel conduction in the pump-like channelrhodopsin ChRmine. *Cell* **185**, 672-689 e623 (2022).
7. Kato H. E.*, et al.* Crystal structure of the channelrhodopsin light-gated cation channel. *Nature* **482**, 369-374 (2012).
8. Volkov O.*, et al.* Structural insights into ion conduction by channelrhodopsin 2. *Science* **358**, eaan8862 (2017).
9. Oda K.*, et al.* Crystal structure of the red light-activated channelrhodopsin Chrimson. *Nat Commun* **9**, 3949 (2018).
10. Nagel G.*, et al.* Channelrhodopsin-2, a directly light-gated cation-selective membrane channel. *Proc Natl Acad Sci U S A* **100**, 13940-13945 (2003).
11. Wietek J., Beltramo, R., Scanziani, M., Hegemann, P., Oertner, T. G. & Wiegert, J. S. An improved chloride-conducting channelrhodopsin for light-induced inhibition of neuronal activity in vivo. *Sci Rep* **5**, 14807 (2015).
12. Tajima S.*, et al.* Structural basis for ion selectivity in potassium-selective channelrhodopsins. Preprint at https://www.biorxiv.org/content/10.1101/2022.10.30.514430v1 (2022).
13. Govorunova E. G.*, et al.* Kalium channelrhodopsins are natural light-gated potassium channels that mediate optogenetic inhibition. *Nat Neurosci* **25**, 967-974 (2022).
